# No influence of patient age on operative treatment outcome of osteochondral lesions of the talus: data from the German Cartilage Registry (GCR, KnorpelRegister DGOU)

**DOI:** 10.1007/s00402-025-05770-7

**Published:** 2025-02-01

**Authors:** Alena Richter, Anna Altemeier, Christoph Becher, Sarah Ettinger, Marco Güllmann, Christian Plaass

**Affiliations:** 1 Diakovere Annastift, Orthopedic Clinic of Hanover Medical School, Hannover, Germany; 2 University Hospital for Orthopaedic and Trauma Surgery, Pius Hospital Oldenburg, Oldenburg, Germany; 3 International Center for Orthopedics at the ATOS Clinic Heidelberg, Heidelberg, Germany; 4 Ruhr University Bochum, Bochum, Germany

**Keywords:** Osteochondral lesion, Talus, Ankle, Age, Registry, Surgical therapy

## Abstract

**Introduction:**

The influence of patient age on the clinical outcome of surgically treated osteochondral lesions of the talus (OCT) is controversial. Aim of this study was to evaluate the 24 months follow-up data of the German Cartilage Registry (KnorpelRegister DGOU, GCR) regarding the influence of patient age on clinical outcomes after surgical OCT treatment.

**Materials and methods:**

303 patients met the inclusion criteria and were divided into patients < 40 years (27.1 ± 5.8 years, *n* = 177) and patients ≥ 40 years (50.8 ± 7.4 years, *n* = 126). Pre- and postoperative FAOS total scores, subscores and ΔFAOS for most frequent surgical techniques (bone marrow stimulation, matrix-augmented bone marrow stimulation, matrix-augmented bone marrow stimulation with additional bone grafting) and lesion size characteristics were evaluated for both groups. ANOVA analysis with post hoc Duncan test was applied for statistical analysis.

**Results:**

Both patients < 40 years and patients ≥ 40 years benefit from surgical treatment of OCT showing significant changes from pre- to postoperative FAOS total score (63.8 ± 20.3 to 81.5 ± 17.8 in patients < 40 years, *p* < 0.001; 57.3 ± 20.1 to 74.9 ± 21.6 in patients ≥ 40 years, *p* < 0.001) and subscores. Younger patient group tended to higher pre- and postoperative scores. ΔFAOS was not different between both groups. Older patient group had significantly higher lesion size area and volume; proportion of additional bone grafting was increased.

**Conclusion:**

Results of surgical therapy of OCTs are independent from patient age. There is no superiority of a specific surgical technique depending on patient age.

**Supplementary Information:**

The online version contains supplementary material available at 10.1007/s00402-025-05770-7.

## Introduction

Osteochondral lesions of the talus (OCT) affect patients at all ages and are a common cause of ankle pain. Although the etiology of OCT is not well understood, juvenile forms, acute traumatic lesions, chronic ankle instability and hindfoot malalignment have been identified as potential cause [1, 2]. OCT represent a relevant clinically and social economically relevant burden [3–6]. Various surgical treatment techniques are described having an overall efficacy rate of 82% [7]. Outcome predictors for the treatment of OCT have been described before [8–10] but the influence of patient age on the clinical outcome remains controversial with studies showing no influence of patient age [11–13] while others detected higher patient age as an independent risk factor for poor outcomes [14, 15]. An age limit predicting worse outcomes after osteochondral therapy ranging between 33 years [15] and 40 years has been shown [8].

Several changes both in biomechanics of the ankle joint [16] and in cellular activity of chondral and bone tissue with increasing age have been described [17, 18].

Former studies do not describe the actual treatment algorithms and are limited to a defined study population, thus not reflecting real life treatment patterns [19]. A differentiated analysis of clinical outcomes of different surgical techniques in different age groups is necessary since defect characteristics has been shown to differ in different age groups requiring specific treatment methods [20].

Therefore, we evaluated data from the German Cartilage Registry (GCR, Deutsches Knorpelregister, DGOU), an observational, nationwide, longitudinal multicenter registry enabling the analysis of real-life treatment patterns and clinical outcomes of cartilage repair techniques of the hip, knee and ankle [21–23]. The outcome of different treatment options of OCT was analyzed depending on patient age.

## Materials and methods

### Registry data

The used data are based on registry data obtained from the GCR. From the introduction of the ankle module in 2015 until the time of data collection in June 2021 the database included 940 patients. Registry data collection is conducted by a web-based Remote Data Entry System. Patients undergoing surgical treatments of ankle OCT are enclosed after written consent. Prospectively, patient data, lesion characteristics, symptoms and outcomes are recorded at specific time points.

## Study design

For this prospective study the following inclusion criteria have been applied to patients recorded in the GCR until June 2021: an isolated osteochondral lesion of the talus (OCT); a completed FAOS subscale pain score 24 month postoperatively as primary outcome parameter; a completely described defect size. There were no specific exclusion criteria once all inclusion criteria were met. Patients were categorized into a group up to 39 years and aged 40 years and older.

## Outcome measures

The in German language edited FAOS score has been used as clinical outcome parameter with FAOS subscale Pain at 24 months follow-up as primary outcome parameter. Preoperative scores and scores at 2 year follow-up have been included. Beside comparison of absolute score values, also the difference between presurgical and postsurgical score values (ΔFAOS) has been analyzed between the different age groups. A further analysis of possible confounding factors on FAOS total score and subscores was performed, including most frequent surgical techniques (i.e. bone marrow stimulation, matrix-augmented bone marrow stimulation and matrix-augmented bone marrow stimulation with additional bone grafting), defect size area and defect volume, BMI and duration of symptoms.

Defects were characterized by area, depth and volume. For volume calculation the ellipsoid volume formula V = (4π/3)*abc, where *a* = length/2, *b* = width/2, and *c* = depth/2, according to Kuni et al. was used [24].

### Statistical analysis

Statistical analysis was performed using SPSS software (version 28.0.0; SPSS Inc., Chicago, IL, USA). All outcome parameters were tested for normal distribution and means and standard deviations were calculated for continuous variables. To detect differences between age groups and treatment modalities a univariate t test (ANOVA) was applied with a post hoc Duncan test. Also patient and lesion characteristics as possible confounding parameters (i.e. BMI, symptom duration, lesion size area, lesion size volume) have been evaluated for both study groups and tested for significant differences as described before. Level of significance was set at 95%. Tested comparisons with a p-level < 0.05 were considered to be significantly different.

## Ethical agreement

GCR is implemented with accordance of nationwide local ethic committees and in consent with the Declaration of Helsinki. All participants provided written consent prior to inclusion in the registry.

## Results

### Patient characteristics

Until June 2021 the ankle module of the GCR included 940 patients. These patients were screened for single OCT (*n* = 867), complete information on defect size (*n* = 813) and complete FAOS subscale pain after 24 month (*n* = 303). Overall, 303 patients met the inclusion criteria and were divided into two age groups, resulting in 177 patients younger than 40 years (27.1 ± 5.8 years) and 126 patients aged 40 years or older (50.8 ± 7.4 years). Mean age was significantly different between both groups (*p* < 0.001). There was no difference regarding other demographic data beside a higher BMI in the older age group (26.0 ± 4.8 kg/m^2^ vs. 28.2 ± 5.2 kg/m^2^, *p* < 0.001) (Table 1).

## Defect characteristics

Significant differences were detected in defect size area and defect size volume (ellipsoid and LxWxH) between younger and older patients. Defect size area measured 123.9 ± 83.7 mm^2^ for patients younger than 40 years and 147.7 ± 110.3 mm^2^ for patients aged 40 years and older (*p* = 0.033). Defect size volume (ellipsoid) was also significantly higher in older patients (381.4 ± 469.3 mm^2^ vs. 537.9 ± 741.5 mm^2^; *p* = 0.025). Defect depth tended to be greater in older patients. However, this difference was not statistically significant (5.0 ± 3.3 mm vs. 5.9 ± 4.6 mm, *p* = 0.051). Symptom duration was similar in both groups (26.8 ± 37.4 months vs. 35.3 ± 52.7 months, *p* = 0.105) (Table 1).

**Table 1:** Demographics and characteristics of our patient cohorts and lesion characteristics

Characteristics	Total	Age < 40	Age ≥ 40	*p*-value
Age at time of surgery	37.0 ± 13.4 (18.0–77.0)	27.1 ± 5.8 (18.0–39.0)	50.8 ± 7.4 (40.0–77.0)	**< 0.001**
Sex				
Male	149 (49.2)	83 (46.9)	66 (52.4)	
Female	154 (50.8)	94 (53.1)	60 (47.6)	
BMI (kg/m2)	26.9 ± 5.1 (16.9–51.3)	26.0 ± 4.8 (16.9–48.2)	28.2 ± 5.2 (18.5–51.3)	**< 0.001**
Duration of Symptoms [month]	30.3 ± 44.5 (0.0-336.0)	26.8 ± 37.4 (0.00-336.00)	35.3 ± 52.7 (2.0-288.0)	0.105
Previous surgery on the respective ankle				
None	191 (63.0)	116 (65.5)	75 (59.5)	
1	82 (27.1)	45 (25.4)	37 (29.3)	
≥ 2	30 (9.9)	16 (9.0)	14 (11.1)	
Previous surgery on OLT				
No	225 (74.3)	133 (75.1)	92 (74.2)	
Yes	76 (25.1)	44 (24.9)	32 (25.8)	
Lesion size area [Length x width (mm²)]	133.8 ± 96.2 (4.0-600.0)	122.9 ± 83.7 (9.0-580.0)	147.9 ± 110.3 (4.0-600.0)	**0.033**
Lesion volume [Length x width x height (mm³)]	852.7 ± 1149.0 (4.0-10450.0)	728.4 ± 896.3 (15.0-7168.0)	1027.3 ± 1416.2 (4.0-10450.0)	**0.025**
Lesion volume [ ellipsoid (mm³)]	446.5 ± 601.6 (2.1-5471.6)	381.4 ± 469.3 (7.9-3753.2)	537.9 ± 741.5 (2.1-5471.6)	**0.025**
Defect depth (mm)	5.4 ± 3.9 (1.0–25.0)	5,0 ± 3,3 (1.0–16.0)	5,9 ± 4,6 (1.0–25.0)	0.051

### Pre- and postoperative FAOS values

**Fig. 1:**
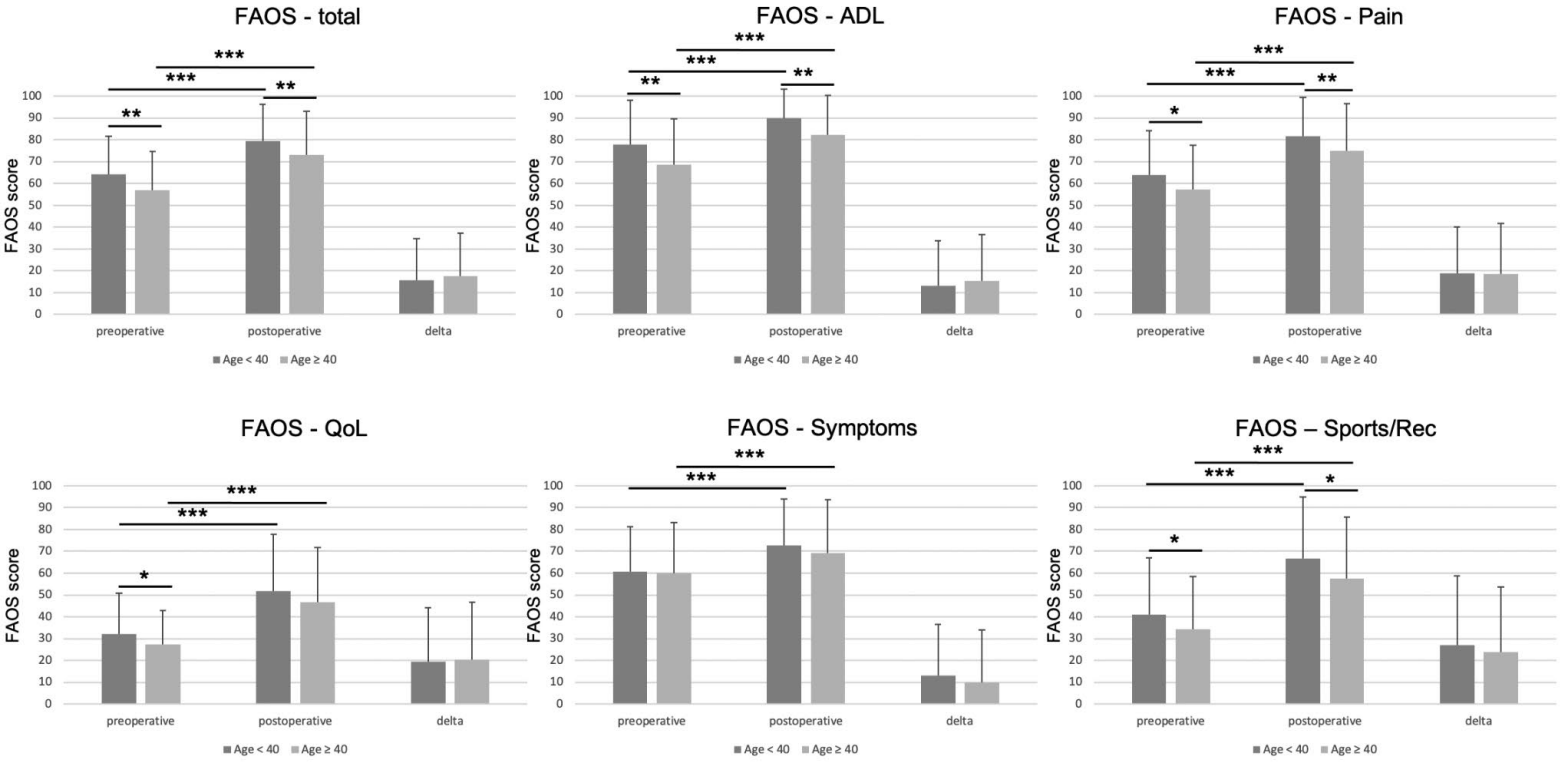
Results of the FAOS total scores, subscales pre- and 24 months postoperatively and ΔFAOS in patients < 40 years (dark grey) and patients ≥ 40 years (light grey). ADL = Activities of daily living; QoL = Quality of Life; Sports/Rec = Sports and Rec (Sports and recreational activities). * *p* < 0.05; ** *p* < 0.01; *** *p* < 0.001

FAOS subscale Pain showed significant changes from pre- to 24 month postoperative in both age groups with an increase from 63.8 ± 20.3 to 81.5 ± 17.8 in patients younger than 40 years and from 57.3 ± 20.1 to 74.9 ± 21.6 in patients aged 40 years and older (*p* < 0.001). Preoperative and postoperative FAOS Pain scores were significantly different between both age groups with lower scores in the older age group (63.8 ± 20.3 vs. 57.3 ± 20.1, *p* = 0.014; 81.5 ± 17.8 vs. 74.9 ± 21.6, *p* = 0.004). The difference between pre- and postoperative scores (ΔFAOS) was not significantly different between both groups (18.7 ± 21.4 vs. 18.6 ± 23.1, *p* = 0.975) (Supplemental material S1, Fig. 1).

Similarly, FAOS total score increased significantly pre- to postoperatively in both age groups. Patients younger than 40 years had significantly higher values both preoperatively (64.3 ± 17.3 vs. 56.8 ± 17.7, *p* = 0.003) and postoperatively (79.7 ± 16.7 vs. 72.9 ± 20.2, *p* = 0.007) compared to patients older than 40 years (Supplemental material S1, Fig. 1).

Significant changes from pre- to postoperative values have been documented both for patients < 40 years and patients ≥ 40 years in all subscales. Patients in the younger age group scored significantly higher in subscales ADL (Activities of daily living; *p* = 0.001), QoL (Quality of live; *p* = 0.034) and Sports/Rec (Sports and recreational activities; *p* = 0.044) preoperatively and in subscales ADL (*p* > 0.001) and Sports/Rec (*p* = 0.007) postoperatively. The amount of pre- to postoperative changes in FAOS scores (ΔFAOS) was not significant different between both groups (Supplemental material S1, Fig. 1).

### Analysis of treatment techniques

**Fig. 2:**
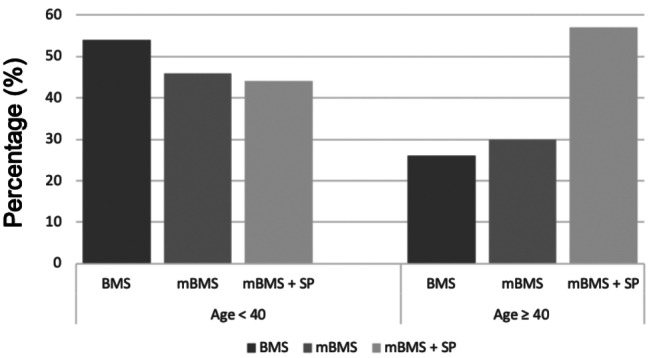
Proportion of most surgical techniques in patients < 40 years and patients ≥ 40 years. BMS (dark grey) = Bone marrow stimulation; mBMS (medium grey) = Matrix-augmented bone marrow stimulation; mBMS + SP (light grey) = Matrix-augmented bone marrow stimulation with additional bone grafting/spongiosa plasty

Most frequent surgical techniques have been bone marrow stimulation (BMS) (31.1%), matrix-augmented bone marrow stimulation (mBMS) (29.6%) and mBMS with additional bone grafting (mBMS + SP) (39.3%). mBMS + SP has been performed more frequently in older patients compared to younger patients. (Fig. 2).

**Fig. 3:**
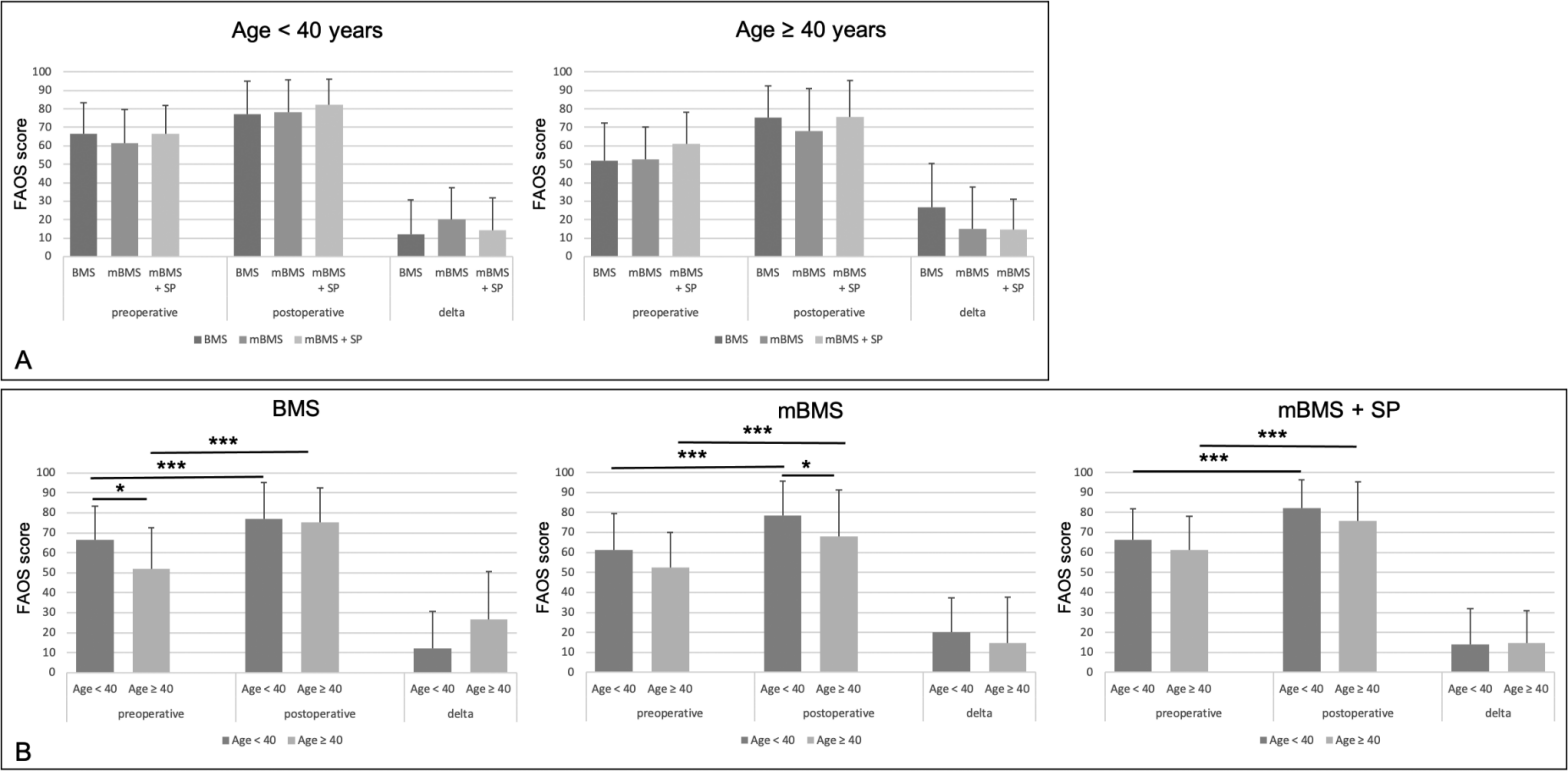
**(A)** Results of the FAOS total scores pre- and 24 months postoperatively and ΔFAOS in patients < 40 years and patients ≥ 40 years with respect to most frequent surgical techniques. BMS (dark grey) = Bone marrow stimulation; mBMS (medium grey) = Matrix-augmented bone marrow stimulation; mBMS + SP (light grey) = Matrix-augmented bone marrow stimulation with additional bone grafting/spongiosa plasty. **(B)** Results of the FAOS total scores pre- and 24 months postoperatively and ΔFAOS in patients < 40 years (dark grey) and patients ≥ 40 years (light grey) after a specific treatment technique. BMS = Bone marrow stimulation; mBMS = Matrix-augmented bone marrow stimulation; mBMS + SP = Matrix-augmented bone marrow stimulation with additional bone grafting/spongiosa plasty. * *p* < 0.05; *** *p* < 0.001

Within the different age cohorts no statistically relevant difference in FAOS total score has been observed between the three surgical techniques (Fig. 3A).

### BMS

BMS was applied in 54 patients < 40 years (44.8%) and in 26 patients ≥ 40 years (23.0%).

Patients younger than 40 years treated with BMS showed significant higher preoperative scores in FAOS total score (66.6 ± 16.9 vs. 52.0 ± 20.5, *p* = 0.010), subscale ADL (81.7 ± 20.5 vs. 66.9 ± 23.3, *p* = 0.017) and Pain (65.4 ± 17.7 vs. 53.2 ± 23.0, *p* = 0.026) than patients aged 40 years or older. Postoperative scores were not significant different in these subscales and total score. Also ΔFAOS did not significantly differ for subscales ADL and Pain, but for FAOS total score with significantly higher change in patients ≥ 40 years (12.0 ± 18.6 vs. 26.6 ± 23.9, *p* = 0.043). Other subscales did not show any statistical differences between both age groups (Supplemental material S 2).

### mBMS

mBMS was applied in 46 patients < 40 years (31.9%) and in 30 patients ≥ 40 years (26.5%).

Significant higher scores in FAOS subscale ADL in patients younger than 40 years compared to the older group was found for mBMS treatment pre- (74.8 ± 22.0 vs. 60.2 ± 23.9, *p* = 0.024) and postoperatively (89.9 ± 13.6 vs. 78.2 ± 22.9, *p* = 0.009). FAOS subscale Pain and Sports/Rec showed statistically higher postoperative scores in the younger group (Pain: 83.0 ± 17.2 vs. 70.0 ± 23.9, *p* = 0.007; Sports: 66.4 ± 28.1 vs. 50.2 ± 30.6, *p* = 0.023) resulting in a higher FAOS total score at 2 year follow-up for patients < 40 years (20.2 ± 17.2 vs. 14.8 ± 22.8, *p* = 0.039). ΔFAOS was not significantly different between both groups both for total score and for subscores (Supplemental material S 3).

### mBMS and additional bone grafting

mBMS with additional bone grafting was applied in 44 patients < 40 years (30.6%) and in 57 patients ≥ 40 years (50.4%).

Patients < 40 years showed significantly higher values for FAOS subscale ADL postoperatively (90.6 ± 13.7 vs. 83.6 ± 17.8, *p* = 0.049) and Pain preoperatively (67.6 ± 17.3 vs. 59.4 ± 17.7, *p* = 0.030) compared to patients ≥ 40 years. FAOS total score, other subscales and ΔFAOS were not statistically different between younger and older patients treated with mBMS + SP (Supplemental material S 4).

## Discussion

The three most important findings of the study were the following: (I) Patients at all ages suffering from single OCT benefit from surgical therapy with significant improvements from pre- to postoperative FAOS scores; (II) Patients < 40 years tended to higher pre- and postoperative scores compared to patients ≥ 40 years reaching significance in some subscores. ΔFAOS was not different between both groups reflecting the potential of surgical therapy independently of patient age; (III) Patients ≥ 40 years showed significantly higher defect size area and defect size volume compared to the younger group.

Since OCTs are very common affecting patients at all age groups, influence of patient age on surgical outcome is important to know for treatment improvement and patient management. Analyzing data of the ankle module of the GCR enables an evaluation of the status quo and identification of influencing factors for clinical outcomes [25]. The difference between registry data and study data has been widely discussed, showing an insufficient representation of the population with cartilage defects in Randomized Controlled Trials (RCTs) [26–28]. Conversely, registry data provide real life treatment patterns and outcomes within a heterogenous population. Data collection was performed prospectively using a web-based Remote Data Entry System asking the 42 items FAOS questionnaire. FAOS score has been used as clinical outcome parameter being validated in German language and for evaluation of OCT before [29, 30].

Patient age has been discussed controversially in the literature before with some studies reporting worse outcomes in older patients and other studies finding no correlation between surgical outcome of OCTs and patient age. Thereby, an age-dependent threshold is hard to find since biological and calendar age can differ considerably. Nevertheless, there seems to be a determining threshold between 30 and 40 years influencing the surgical outcome of OCTs. Accordingly, Chuckpaiwong et al. [8] detected a mean age of 38.6 years in patients with better outcomes and a mean age of 44.1 years in patients with worse outcomes after BMS of OCT. Cuttica et al. [15] showed an increasing risk for worse outcomes after BMS with increasing age up to a limit of 33 years. Behind this threshold, age did not influence the surgical outcome anymore. Similarly, in knee surgery influence of patient age has been examined with a tendency towards poorer outcomes in middle-aged patients between 40 and 60 years compared to younger patients, especially in cell-based therapies comprising autologous chondrocyte implantation (ACI) and bone marrow aspirate concentrate [31]. Furthermore, patients aged 40 years and older had significantly worse outcomes after BMS compared to patients younger than 40 years [32]. In contrast, after augmentation of BMS with a matrix (mBMS) no influence of patient age could be detected [33].

In the current study, patients were divided in two groups, i.e. < 40 years and ≥ 40 years, based on these differences in the outcome of cartilage therapy regarding patient age.

Significant changes from pre- to postsurgical values were found both in younger and older patients achieving an improvement above the minimal clinical important difference (MCID) and the minimal detectable change (MDC) defined for FAOS [29, 34] reflecting the clinical importance for both age groups. Nevertheless, younger patients showed higher absolute pre- and postoperative FAOS values. This was similar to the results of D’Ambrosi et al. [11] and is also supported by findings of Gottschalk et al. concluding that the clinical outcome after cartilage therapy is strongly influenced by the preoperative status [35]. Therefore, an increased level of symptoms in older patients can also be related to general degenerative changes in the ankle.

In some literature, an independent effect of patient age on postsurgical outcome is described [8, 9]. In these studies, only bone marrow stimulation and debridement have been applied. Also Cuttica et al. [15] found inferior outcomes with increasing age up to 33 years after BMS. D’Ambrosi et al. [11] transmitted these results assessing functional outcome after mBMS in two age groups with a cutoff at 33 years. In contrast with the former results, both age groups showed similar healing of OCTs in follow-up confirming effectiveness of mBMS independent of patient age. We therefore analyzed clinical outcomes in different age groups depending on the most applied surgical techniques, i.e. BMS, mBMS and mBMS with additional bone grafting. All these techniques have shown promising results before [36]. Interestingly, we found a marginally significant higher improvement ΔFAOS from preoperative to postoperative FAOS total score 24 months after BMS in patients ≥ 40 years compared to the younger group (12.0 ± 18.6 vs. 26.6 ± 23.9, *p* = 0.043). Nevertheless, postoperative FAOS total score did not differ significantly between both groups. This is in accordance to other studies finding no correlation between patient age and surgical outcome [12, 13].

Subgroup analysis of patients being treated with mBMS revealed significantly higher postoperative FAOS values for the younger patients in subscales ADL, pain and Sports/Rec resulting in higher postoperative total score for patients < 40 years. The amount of improvement did not show any differences. Similarly, additional bone grafting did not lead to different improvements ΔFAOS between both age group.

Comparing the outcomes of these most frequent techniques within both age groups did not reveal any superiority of a specific treatment emphasizing the concept of lesion-dependent technique as recommended in current treatment algorithms [23, 37] and not age-dependent technique. Accordingly, lesion size areas exceeding 1 cm^2^ should be treated by mBMS and in case of lesion depths exceeding 5 mm additional bone grafting is recommended [38–41]. We also analyzed the distribution of applied surgical techniques according to patient age. While BMS distributed equally in the total cohort with nearly 30%, significant differences were found in the older age group with a higher prevalence of mBMS + SP accounting for 50.4% of performed therapies. Because of the different distribution of surgical techniques between both age cohorts defect size as determining factor for therapy choice was analyzed showing a significantly higher defect size area and defect size volume in patients ≥ 40 years. Defect depth was also increased in older patients but nearly did not reach significance. In accordance, different lesion characteristics depending on patient age are described in the literature before revealing a higher incidence of cystic lesions in older patients [20].

Beside lesion characteristics and treatment techniques BMI and symptom duration have been documented and compared between both groups as possible confounding parameters. Mean BMI was significantly higher in patients ≥ 40 years. The influence of BMI on postoperative OCT outcome has been discussed controversially [8, 42, 43] and thus has to be considered as a possible confounder even if there were no significant differences in the improvement of clinical outcome measures at 2 years follow-up between both groups. Symptom duration was not significantly different between patients < 40 years and patients ≥ 40 years.

Overall, the findings of our study encourage the use of operative treatment of focal osteochondral lesions of the ankle at every patient age since significant clinical improvement can be achieved. Since registry data contain real life treatment patterns instead of experimentally performed treatment algorithms it can be assumed that appropriate techniques have been applied depending on defect characteristics stated by general recommendations. This can also be seen as a limitation in analyzing the influence of patient age on different techniques. Our study has some other limitations due to the use of registry data meaning a wide heterogeneity of surgical techniques, surgeon skills, applied materials and postsurgical treatment. Due to limited case numbers and different definitions, we also did not analyze for biomechanical co-factors, like instability or deformity. Furthermore, we did not include adolescent patients younger than 18 years. It is well known that micro fracturing is more beneficial in non-adult patients [9]. Another limitation is a significant difference in mean BMI between the two cohorts with a higher BMI in older patients. Last but not least, only the clinical outcome at 2 year follow up has been analyzed. It would be interesting to investigate earlier time points regarding regeneration time and also long term follow-ups regarding a possible earlier deterioration in older patients.

## Conclusion

Results of surgical therapy of OCTs are independent from patient age. Patients older than 40 years show more symptoms pre- and postoperatively than younger patients. Nevertheless, improvement with significant clinical symptom reduction can be achieved at all ages. Thereby, appropriate choice of surgical technique according to general recommendations seems to be essential. There is no superiority of a specific surgical technique depending on patient age.

## Electronic Supplementary Material

Below is the link to the electronic supplementary material.

Supplemental Material

## Data Availability

No datasets were generated or analysed during the current study.
