# Supplementary material for: No influence of patient age on operative treatment outcome of osteochondral lesions of the talus: data from the German Cartilage Registry (GCR, KnorpelRegister DGOU)
Source: Arch Orthop Trauma Surg. 2025 Feb 1;145(1):151. doi: 10.1007/s00402-025-05770-7 (PMC11787207; doi:10.1007/s00402-025-05770-7)
Supplement: Supplementary file 1 — Supplemental Material [file 402_2025_5770_MOESM1_ESM.docx]

**Supplemental Material**

**S 1: Analysis of FAOS total score and FAOS subscales between both age groups pre- and postoperatively at 24 months follow-up**

| **FAOS-Score** | **Age < 40** | **Age ≥ 40** | **p – value** |
| --- | --- | --- | --- |
| **Activity preop.** | 77.8 ± 20.2 (0.0-100.0) | 68.6 ± 20.9 (4.4-100.0) | **0.001** |
| **Activity postop.** | 89.7 ± 13.5 (30.9-100.0) | 82.1 ± 18.3 (13.2-100.0) | **< 0.001** |
| **Delta Activity** | 13.1 ± 20.5 (-23.5-94.1) | 15.2 ± 21.3 (-19.1-80.9) | 0.479 |
| **Pain preop.** | 63.8 ± 20.3 (0.0-100.0) | 57.3 ± 20.1 (2.8-100.0) | **0.014** |
| **Pain postop.** | 81.5 ± 17.8 (25.0-100.0) | 74.9 ± 21.6 (11.1-100.0) | **0.004** |
| **Delta Pain** | 18.7 ± 21.4 (-36.1-91.7) | 18.6 ± 23.1 (-41.7-72.2) | 0.975 |
| **QoL preop.** | 32.1 ± 18.8 (0.0-93.8) | 27.4 ± 15.5 (0.0-75.0) | **0.034** |
| **QoL postop** | 51.6 ± 26.2 (0.0-100.0) | 46.7 ± 24.9 (0.0-100.0) | 0.106 |
| **Delta QoL** | 19.3 ± 24.9 (-37.5-81.3) | 20.5 ± 26.1 (-25.0-87.5) | 0.716 |
| **Symptoms preop.** | 60.6 ± 20.6 (14.3-100.0) | 60.0 ± 23.0 (14.3-100.0) | 0.831 |
| **Symptoms postop.** | 72.7 ± 21.1 (17.9-100.0) | 69.3 ± 24.3 (10.7-100.0) | 0.193 |
| **Delta Symptoms** | 13.0 ± 23.4 (-39.3-82.1) | 9.9 ± 24.0 (-57.1-78.6) | 0.297 |
| **Sports preop.** | 41.1 ± 26.0 (0.0-100.0) | 34.4 ± 24.1 (0.0-90.0) | **0.044** |
| **Sports postop.** | 66.8 ± 28.1 (0.0-100.0) | 57.5 ± 28.3 (0.0-100.0) | **0.007** |
| **Delta Sports** | 27.0 ± 31.6 (-65.0-100.0) | 24.0 ± 29.6 (-50.0-90.0) | 0.465 |
| **Total preop.** | 64.3 ± 17.3 (11.3-99.4) | 56.8 ± 17.7 (16.7-93.5) | **0.003** |
| **Total postop.** | 79.4 ± 16.7 (30.4-100.0) | 72.9 ± 20.2 (12.5-100.0) | **0.007** |
| **Delta Total** | 15.7 ± 18.9 (-17.9-66.7) | 17.6 ± 19.7 (-19.1-68.5) | 0.518 |

**S 2: Analysis of FAOS total score and FAOS subscales between both age groups pre- and postoperatively at 24 months follow-up after BMS treatment**

| **FAOS-Score** | **Age < 40** | **Age ≥ 40** | **p – value** |
| --- | --- | --- | --- |
| **Activity preop.** | 81.7 ± 20.5 (0.0-100.0) | 66.9 ± 23.3 (26.5-98.5) | **0.017** |
| **Activity postop.** | 88.7 ± 14.0 (47.1-100.0) | 84.3 ± 13.4 (52.9-100.0) | 0.214 |
| **Delta Activity** | 18.6 ± 19.2 (-16.2-94.1) | 17.8 ± 25.2 (-16.2-67.6) | 0.150 |
| **Pain preop.** | 65.4 ± 17.7 (25.0-100.0) | 53.2 ± 23.0 (25.0-88.9) | **0.026** |
| **Pain postop.** | 78.0 ± 19.2 (36.1-100.0) | 78.9 ± 16.3 (52.8-100.0) | 0.853 |
| **Delta Pain** | 14.6 ± 22.4 (-36.1-63.9) | 25.4 ± 24.2 (-13.9-72.2) | 0.091 |
| **QoL preop.** | 32.1 ± 16.8 (0.0 -93.8) | 27.7 ± 17.5 (0.0-75.0) | 0.331 |
| **QoL postop** | 49.7 ± 27.0 (0.0 -100.0) | 44.5 ± 20.3 (12.5-81.3) | 0.387 |
| **Delta QoL** | 18.8 ± 26.9 (-25.0-81.3) | 16.7 ± 22.0 (-18.7-68.8) | 0.760 |
| **Symptoms preop.** | 61.9 ± 20.7 (28.6-100.0) | 59.3 ± 24.2 (17.9-100.0) | 0.647 |
| **Symptoms postop.** | 70.4 ± 21.7 (28,6-100.0) | 70.9 ± 24.5 (28.6-100.0) | 0.931 |
| **Delta Symptoms** | 10.3 ± 23.9 (-32.1-67.9) | 10.2 ± 30.7 (-46.4-78.6) | 0.993 |
| **Sports preop.** | 43.0 ± 22.0 (0.0-100.0) | 38.3 ± 30.3 (0.0-90.0) | 0.493 |
| **Sports postop.** | 66.3 ± 29.1 (0.0-100.0) | 56.1 ± 29.4 (10.0-100.0) | 0.173 |
| **Delta Sports** | 24.9 ± 31.8 (-30.0-100.0) | 20.0 ± 35.3 (-30.0-95.0) | 0.603 |
| **Total preop.** | 66.6 ± 16.9 (11.3-99.4) | 52.0 ± 20.5 (25.0-86.9) | **0.010** |
| **Total postop.** | 77.0 ± 18.0 (41.7-100.0) | 75.4 ± 16.9 (42.3-98.2) | 0.750 |
| **Delta Total** | 12.0 ± 18.6 (-16.7-66.7) | 26.6 ± 23.9 (-8.3-68.5) | **0.043** |

**S 3: Analysis of FAOS total score and FAOS subscales between both age groups pre- and postoperatively at 24 months follow-up after mBMS treatment**

| **FAOS-Score** | **Age < 40** | **Age ≥ 40** | **p – value** |
| --- | --- | --- | --- |
| **Activity preop.** | 74.8 ± 22.0 (30.9-100.0) | 60.2 ± 23.9 (4.4-94.1) | **0.024** |
| **Activity postop.** | 89.9 ± 13.6 (47.1-100.0) | 78.2 ± 22.9 (13.2-100.0) | **0.009** |
| **Delta Activity** | 17.7 ± 20.7 (-11.8-69.1) | 17.4 ± 27.6 (-19.1-80.9) | 0.973 |
| **Pain preop.** | 61.9 ± 20.4 (22.2-100.0) | 54.9 ± 23.1 (2.8-91.7) | 0.239 |
| **Pain postop.** | 83.0 ± 17.2 (25.0-100.0) | 70.0 ± 23.9 (11.1-100.0) | **0.007** |
| **Delta Pain** | 21.2 ± 17.8 (-8.3-66.7) | 14.4 ± 22.8 (-16.7-66.7) | 0.215 |
| **QoL preop.** | 29.2 ± 20.3 (0.0-68.8) | 23.8 ± 14.1 (0.0-62.5) | 0.243 |
| **QoL postop** | 53.1 ± 28.6 (0.0-100.0) | 44.2 ± 27.8 (0.0-100.0) | 0.185 |
| **Delta QoL** | 23.4 ± 25.5 (-18.7-75.0) | 19.5 ± 26.2 (-18.7-68.8) | 0.564 |
| **Symptoms preop.** | 57.9 ± 21.5 (14.3-92.9) | 52.7 ± 21.1 (14.3-92.9) | 0.339 |
| **Symptoms postop.** | 71.4 ± 21.2 (21.4-100.0) | 63.6 ± 25.0 (10.7-100.0) | 0.145 |
| **Delta Symptoms** | 14.6 ±21.4 (-28.6-67.9) | 11.4 ± 17.3 (-21.4-39.3) | 0.529 |
| **Sports preop.** | 34.0 ± 27.4 (0.0-100.0) | 26.1 ± 18.8 (0.0-60.0) | 0.239 |
| **Sports postop.** | 66.4 ± 28.1 (5.0-100.0) | 50.2 ± 30.6 (0.0-100.0) | **0.023** |
| **Delta Sports** | 34.4 ± 25.8 (-5.0-100.0) | 22.9 ± 35.1 (-50.0-95.0) | 0.170 |
| **Total preop.** | 61.3 ± 18.2 (25.6-92.3) | 52.5 ± 17.5 (16.7-79.8) | 0.103 |
| **Total postop.** | 78.3 ± 17.3 (30.4-99.4) | 67.9 ± 23.2 (12.5-100.0) | **0.039** |
| **Delta Total** | 20.2 ± 17.2 (-1.8-61.3) | 14.8 ± 22.8 (-19.0-60.7) | 0.383 |

**S 4: Analysis of FAOS total score and FAOS subscales between both age groups pre- and postoperatively at 24 months follow-up after mBMS with additional bone grafting**

| **FAOS-Score** | **Age < 40** | **Age ≥ 40** | **p – value** |
| --- | --- | --- | --- |
| **Activity preop.** | 79.1 ± 16.3 (41.2-100.0) | 72.7 ± 18.7 (29.4-100.0) | 0.104 |
| **Activity postop.** | 90.6 ± 13.7 (30.9-100.0) | 83.6 ± 17.8 (42.6-100.0) | **0.049** |
| **Delta Activity** | 9.9 ± 16.4 (-23.5-52.9) | 12.8 ± 16.7 (-17.6-58.8) | 0.478 |
| **Pain preop.** | 67.6 ± 17.3 (25.0-94.4) | 59.4 ± 17.7 (22.2-100.0) | **0.030** |
| **Pain postop.** | 83.0 ± 16.3 (38.9-100.0) | 75.9 ± 22.3 (27.8-100.0) | 0.081 |
| **Delta Pain** | 16.7 ± 17.5 (-19.4-52.8) | 18.4 ± 21.6 (-27.8-69.4) | 0.693 |
| **QoL preop.** | 33.2 ± 19.4 (0.0-81.3) | 28.4 ± 15.5 (0.0-62.5) | 0.189 |
| **QoL postop** | 50.9 ± 23.4 (6.3-93.8) | 48.8 ± 26.4 (0.0-100.0) | 0.682 |
| **Delta QoL** | 18.6 ± 23.2 (-37.5-75.0) | 23.0 ± 28.3 (-25.0-87.5) | 0.428 |
| **Symptoms preop.** | 61.9 ± 20.1 (17.9-100.0) | 64.9 ± 23.3 (14.3-100.0) | 0.521 |
| **Symptoms postop.** | 76.3 ± 17.2 (32.1-100.0) | 72.2 ± 24.2 (10.7-100.0) | 0.342 |
| **Delta Symptoms** | 15.4 ± 20.8 (-28.6-46.4) | 8.3 ± 24.3 (-57.1-57.1) | 0.142 |
| **Sports preop.** | 45.1 ± 28.4 (0.0-100.0) | 37.6 ± 23.2 (0.0-85.0) | 0.174 |
| **Sports postop.** | 66.5 ± 27.5 (10.0-100.0) | 62.7 ± 27.2 (0.0-100.0) | 0.497 |
| **Delta Sports** | 22.3 ± 31.1 (-65.0-85.0) | 25.1 ± 23.1 (-20.0-90.0) | 0.640 |
| **Total preop.** | 66.4 ± 15.5 (40.5-95.8) | 61.2 ± 16.9 (23.2-93.5) | 0.174 |
| **Total postop.** | 82.1 ± 14.2 (34.5-98.8) | 75.6 ± 19.8 (32.7-100.0) | 0.107 |
| **Delta Total** | 14.1 ± 17.7 (-17.9-45.8) | 14.6 ± 16.3 (-15.5-64.3) | 0.910 |
